# Primary cilia on muscle stem cells are critical to maintain regenerative capacity and are lost during aging

**DOI:** 10.1038/s41467-022-29150-6

**Published:** 2022-03-17

**Authors:** Adelaida R. Palla, Keren I. Hilgendorf, Ann V. Yang, Jaclyn P. Kerr, Aaron C. Hinken, Janos Demeter, Peggy Kraft, Nancie A. Mooney, Nora Yucel, David M. Burns, Yu Xin Wang, Peter K. Jackson, Helen M. Blau

**Affiliations:** 1 Blau Laboratory, Baxter Laboratory for Stem Cell Biology, Department of Microbiology and Immunology, Institute for Stem Cell Biology and Regenerative Medicine, Stanford School of Medicine, Stanford, CA 94305-5175 USA; 2 Jackson Laboratory, Baxter Laboratory for Stem Cell Biology, Department of Microbiology and Immunology, Institute for Stem Cell Biology and Regenerative Medicine, Stanford School of Medicine, Stanford, CA 94305-5175 USA; 3 GlaxoSmithKline Research and Development, Muscle Metabolism Discovery Performance Unit, 1250S. Collegeville Rd., Collegeville, PA 19426 USA; 4 Department of Pathology, Stanford University School of Medicine, Stanford, CA 94305 USA; 5 Present Address: Department of Biochemistry, University of Utah School of Medicine, Salt Lake City, UT 84112 USA

**Keywords:** Cilia, Ageing, Muscle stem cells

## Abstract

During aging, the regenerative capacity of muscle stem cells (MuSCs) decreases, diminishing the ability of muscle to repair following injury. We found that the ability of MuSCs to regenerate is regulated by the primary cilium, a cellular protrusion that serves as a sensitive sensory organelle. Abolishing MuSC cilia inhibited MuSC proliferation in vitro and severely impaired injury-induced muscle regeneration in vivo. In aged muscle, a cell intrinsic defect in MuSC ciliation was associated with the decrease in regenerative capacity. Exogenous activation of Hedgehog signaling, known to be localized in the primary cilium, promoted MuSC expansion, both in vitro and in vivo. Delivery of the small molecule Smoothened agonist (SAG1.3) to muscles of aged mice restored regenerative capacity leading to increased strength post-injury. These findings provide fresh insights into the signaling dysfunction in aged MuSCs and identify the ciliary Hedgehog signaling pathway as a potential therapeutic target to counter the loss of muscle regenerative capacity which accompanies aging.

## Abstract (web summary)

Repair of muscle damage requires muscle stem cells, which lose regenerative capacity with aging. Here, the authors show that a sensory organelle, the primary cilium, is critical for muscle stem cell proliferation during regeneration and lost with aging.

## Introduction

Adult muscle stem cells (MuSCs), also known as satellite cells, are crucial for skeletal muscle regeneration throughout life^1^. Sarcopenia, the age-dependent loss of skeletal muscle mass and strength, is a major public-health problem that affects an estimated 15% of individuals 65 years or older^2,3^. With aging, MuSCs undergo intrinsic changes that reduce their number and function, which decreases the capacity of muscle to respond to exercise and injury and efficiently repair damage to muscle fibers^1^. Such MuSC intrinsic changes include atypical activation of p38-MAPK or Jak2-Stat3 signaling^4–8^. Additionally, extrinsic changes that alter the niche, or MuSC microenvironment, impact MuSC regenerative function with aging^5^. Finally, maintenance of the quiescent cell state is essential for muscle stem cell function^9^. With aging, the increase in cytokines in muscles, including FGF2, WNT3A and TGF-β alter the signaling state of aged MuSCs leading to their premature exit from quiescence^5,9–11^. Despite this knowledge, our understanding of the basis for MuSC dysfunction with aging remains incomplete.

Many adult stem cells possess a primary cilium, a microtubule-based, antenna-like structure that protrudes from the cell surface to mediate sensory and morphogenetic signaling^12^. Primary cilia transduce signaling from a growing list of G-protein coupled receptors (GPCRs), proteins with seven transmembrane helices capable of transducing extracellular stimuli into intracellular signals. GPCRs are of particular interest, as they constitute a potent class of drug targets^13^. A recent report showed that MuSCs and muscle progenitor cells possess a primary cilium that is linked to their cell cycle state^14,15^, but the role of primary cilia in MuSC function in vivo has yet to be established.

One of the most characterized ciliary signaling pathways is Hedgehog (Hh) signaling, critical for both embryonic development and tissue homeostasis and regeneration. There are three known mammalian Hh ligands, Sonic-Hedgehog, Indian-Hedgehog, and Desert-Hedgehog. The primary cilium is required to organize canonical signaling by all three ligands in most vertebrate cells^16^. Briefly, in the absence of Hh ligand, its receptor Patched (PTCH1) is localized to and around the primary cilium and downstream GLI transcription factors are proteolytically processed to a repressor form^17,18^. In the presence of Hh ligand, its receptor PTCH1 exits the primary cilium, while the GPCR SMO accumulates in the cilium and active GLI transcription factors are released for translocation to the nucleus and expression of downstream GLI target genes, such as *Ptch1* and *Gli2*^19^. Hh has previously been shown to play a role in myogenesis, especially during embryogenesis, as canonical Hh signaling to somites is critical for the induction of myogenic factors such as MYOD1 and MYF5^20^, and the subsequent induction of slow twitch muscle fates^21^. In muscle progenitor cells, Hh signaling has also been characterized as a pro-survival and proliferation factor^22^.

Here, we show that genetic ablation of primary cilia in adult MuSCs dramatically decreases their self-renewal and regenerative capacity in vivo. Additionally, we identify loss of ciliation as an intrinsic defect of MuSCs with aging, and that augmenting the regenerative capacity of aged MuSCs through activation of the ciliary Hedgehog (Hh) signaling pathway is a previously unrecognized therapeutic strategy for the treatment of sarcopenia.

## Results

### Loss of cilia on MuSCs impairs muscle regeneration and strength recovery

**Fig. 1:**
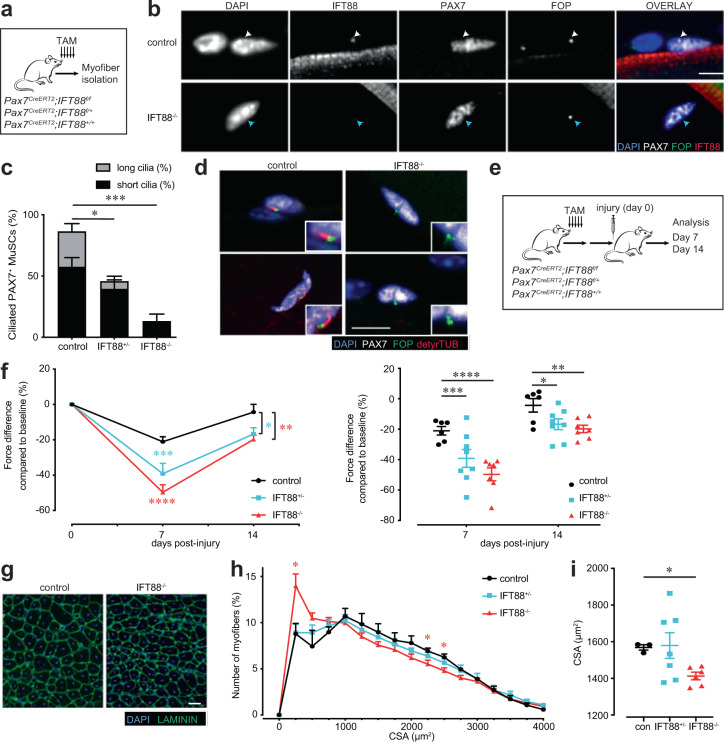
Loss of primary cilia in MuSCs impairs muscle regeneration and strength recovery. **a** Pax7-specific Ift88 conditional knockout mice (*Pax7*^*CreERT2*^*;IFT88*^*f*/*f*^, IFT88^−/−^*)*, heterozygous mice (*Pax7*^*CreERT2*^*;IFT88*^*+/f*^, IFT88^+/−^*)* or control littermates (*Pax7*^*CreERT2*^*;IFT88*^*+/+*^, control, con*)* were treated with tamoxifen (TAM) at 8 weeks of age. Myofibers were isolated and stained for ciliary markers. **b** Representative confocal images of control and IFT88^−/−^ myofibers showing IFT88 and FOP staining in PAX7^+^ MuSCs. All cells stain positive for the centrosomal marker FOP. Scale bars: 10 μm. DAPI, blue; PAX7, white; FOP, green; IFT88, red. White arrowheads indicate the presence of IFT88 in the primary cilium on the surface of MuSCs. Blue arrowheads indicate MuSCs lacking cilia. Quantification in Supplementary Fig. 1b. **c**, **d** Visualization of MuSC cilia using detyrosinated tubulin to stain the ciliary axoneme and FOP to visualize the ciliary base. All cells stain positive for the centrosomal marker FOP. Absence of detyrosinated tubulin is scored as no cilia. **c** Percent of short (<1 µM) and long (>1 µM) cilia on Pax7+ MuSCs quantified from isolated myofibers of control, IFT88^+/−^ and IFT88^−/−^ mice (*n* = 75 control, 79 IFT88^+/−^, 81 IFT88^−/−^ total myofibers were analyzed from 4 independent mice per genotype; average percent of ciliated MuSCs per mouse is shown, individual data points in Supplementary Fig. 1c). **d** Representative confocal images of uninjured/resting EDL myofibers of control and IFT88^−/−^ mice showing cilia immunostaining in Pax7^+^ MuSCs. Scale bars: 10 μm. DAPI, blue; PAX7, white; FOP, green; detyrosinated tubulin, red. **e**–**i** Control, IFT88^+/−^ and IFT88^−/−^ mice were injured with notexin and analysis was performed 7 or 14 days post-injury (*n* = 3 control mice; *n* = 4 IFT88^+/−^ and IFT88^−/−^; two legs per mouse). **e** Experimental scheme. **f** Plantar flexion tetanic torque of control, IFT88^+/−^ and IFT88^−/−^ mice on day 7 and day 14 post-injury (values normalized to baseline torque). **g** Representative *Gastrocnemius* (GA) cross-section at 14 days post-injury from control and IFT88^−/−^ mice. DAPI, blue; LAMININ, green. Bar=50 µm. **h** Myofiber cross-sectional areas (CSA) in control, IFT88^+/−^ and IFT88^−/−^ GAs (*n* = 3 for control, *n* = 6 for IFT88^+/−^ and *n* = 6 for IFT88^−/−^). **i** Mean CSA. *P < 0.05, **P < 0.01, ***P < 0.001 ****P < 0.0001. ANOVA test with Fisher’s LSD test for multiple comparisons (**c**, **f**, **h**, **i**). Source data are provided as a Source Data file. Means+s.e.m.

We first sought to determine if primary cilia in MuSCs are required for their self-renewal and regenerative capacity in response to injury. Pax7 is the hallmark transcription factor expressed by MuSCs^23^. We confirmed MuSC ciliation using a transgenic mouse model that expresses fluorescently tagged CENTRIN2 and ARL13B to visualize the base and axoneme of primary cilia, respectively^24,25^ (Supplementary Fig. 1a). To assess the functional significance of MuSC ciliation, we generated a Pax7^CreERT2^;IFT88^f/f^ mouse model in which the intraflagellar transport protein IFT88, required for ciliary assembly and maintenance^26^, is specifically and conditionally ablated in Pax7-expressing MuSCs (Fig. 1a, b and Supplementary Fig. 1b). We assessed cilia of PAX7-positive MuSCs using antibodies directed against detyrosinated tubulin to visualize the ciliary axoneme and the centriolar marker FGFR1OP/FOP to visualize the ciliary base. Henceforth, we define percent ciliation as the percentage of cells staining positive for detyrosinated tubulin. We confirmed a decrease in the percent and length of ciliation in tamoxifen-treated Pax7^CreERT2^; IFT88^f/f^ (IFT88^−/−^) MuSCs compared to littermate Pax7^CreERT2^;IFT88^+/+^ controls (control) on isolated myofibers by immunofluorescence microscopy (Fig. 1c, d and Supplementary Fig. 1c). Of note, heterozygous Pax7^CreERT2^;IFT88^+/f^ (IFT88^+/−^) MuSCs exhibited an intermediate decrease in ciliation (Fig. 1c and Supplementary Fig. 1c). To assess the functional consequence of MuSC ciliation loss, we performed notexin-induced injury in the *Gastrocnemius* muscle of young (2–4 months) control, IFT88^+/−^, and IFT88^−/−^ mice and measured strength recovery. Notexin, a widely used injury paradigm, is a phospholipase A2 neurotoxin peptide extracted from snake venom that acts as a myotoxin upon intramuscular injection and promotes myofiber necrosis, but spares muscle stem cells, which become activated and proliferate to regenerate the injured muscle. We found that homozygous loss of IFT88 led to a 50% decrease in tetanic force 7 days post-injury and a 20% decrease at 14 days post-injury compared to baseline, and force was significantly reduced compared to controls (Fig. 1e, f and Supplementary Fig. 1d, e). Similarly, muscle mass was decreased in IFT88^−/−^ mice at day 14 compared to controls (Supplementary Fig. 1f). Regeneration potential was assessed by histological examination of injured muscles. To regenerate injured muscles, MuSCs must become activated and proliferate to generate a pool of myoblasts, which differentiate and fuse to form new myofibers or replenish existing myofibers to repair and replace the injured area^27^. A shift toward myofibers with reduced cross-sectional area (CSA), as well as reduced mean CSA, at day 14 post-injury quantified by immunofluorescence was observed, indicative of either impaired or delayed regenerative capacity of IFT88^−/−^ MuSCs (Fig. 1g-i). The intermediate regenerative capacity of IFT88^+/−^ MuSCs is consistent with the partial loss of ciliation observed in MuSCs from heterozygous mice (Fig. 1c, f and Supplementary Fig. 1b–e). These results demonstrate that in the absence of the primary cilium on MuSCs, muscle regeneration is impaired, and force is not fully restored after notexin-induced injury.

### Primary cilia on MuSCs are required for MuSC proliferation and engraftment

**Fig. 2:**
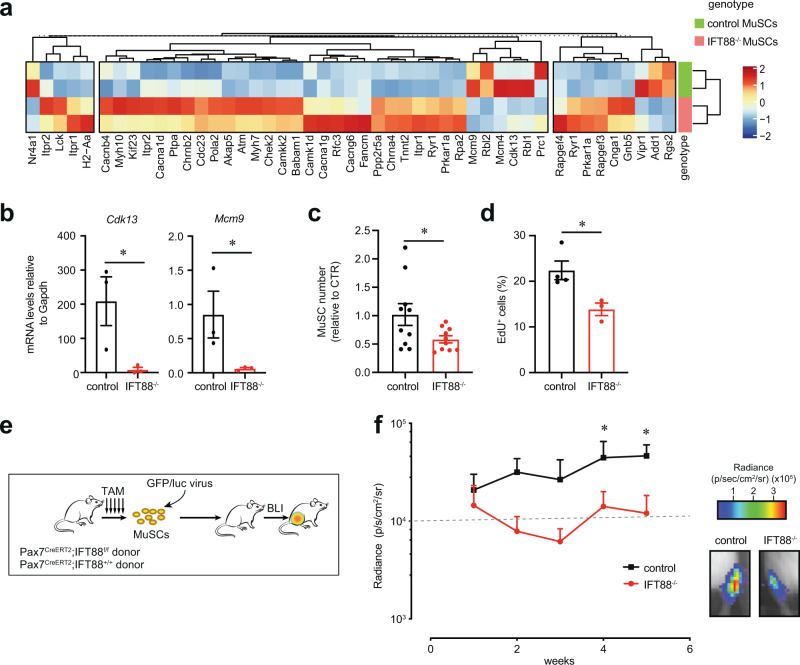
Loss of primary cilia in MuSCs reduces proliferative and self-renewal capacity. **a** Heat map of differentially expressed genes (*p* < 0.1) in IPA (Ingenuity Pathway Analysis) enriched pathways between control or IFT88^−/−^ MuSCs freshly isolated from hindlimb muscles. **b** Expression of cell cycle genes *Cdk13* and *Mcm9* is decreased in IFT88^−/−^ MuSCs compared to controls as determined by qPCR (*n* = 3 mice per condition). **c** Number of control and IFT88^−/−^ MuSCs after culture on hydrogel for 7 days, normalized to control (*n* = 10 mice per group performed in 3 independent experiments). **d** Percentage of EdU^+^ MuSCs (*n* = 4 mice for control, *n* = 3 mice for IFT88^−/−^). **e**, **f** Engraftment of GFP/luc-labeled control and IFT88^−/−^ MuSCs. **e** Transplant scheme. **f** Left: Bioluminescence imaging (BLI) signal post-transplant expressed as average radiance (p s^−1^ cm^−2^ sr^−1^) (*n* = 5 replicates per condition, individual data points in Supplementary Fig. 2f). Right: Representative BLI images for each condition. Mann–Whitney test (**b**–**d**). 2 way ANOVA test with Fisher’s LSD for multiple comparisons per time point (**f**). *P < 0.05, **P < 0.01. Source data are provided as a Source Data file. Means+s.e.m.

To elucidate the role of primary cilia in maintaining MuSC function, we performed RNAseq analyses of control and IFT88^−/−^ MuSCs. Pathways related to calcium signaling (i.e*. Akap5, Cacna1d, Cacna1g, Cacnab, Camk1d, Camkk2, Chrna4, Chrnb2*), Gα signaling (i.e*. Add1, Cnga1, Gnb5, Prkar1a, Rapgef4, Rapgef4, Rgs2*) and cell cycle (i.e*. Atm, Chek2, Babam1, Cdk13, Mcm9*) were differentially expressed in IFT88^−/−^ and control MuSCs (Fig. 2a and Supplementary Fig. 2a, b, Supplementary Data 1) in good agreement with the function of primary cilia in other cell types^25,28–30^.

We confirmed by qPCR that expression of cell cycle genes, *Cdk13* and *Mcm9*, was decreased in IFT88^−/−^ MuSCs compared to controls (Fig. 2b). To analyze the proliferative potential of MuSCs, we plated them on elastic hydrogels with a stiffness equivalent to that of muscle tissue (12 kPa), which preserves their self-renewal properties in culture^31^. IFT88^−/−^ MuSCs exhibited a reduced proliferative capacity compared to controls, as determined by the total number of MuSCs after 7 days (Fig. 2c). We further confirmed this reduction in proliferative capacity using an EdU incorporation assay (Fig. 2d). We observed a similar phenotype when we performed knockdown of other ciliary components which are essential for cilia assembly, *Kif3a* and *Cep164* (Supplementary Fig. 2c, d), confirming the role of primary cilia in MuSC proliferation. To elucidate if loss of ciliary signaling and consequent reduction in MuSC proliferation could result from spontaneous differentiation of MuSCs, we plated control and IFT88^−/−^ MuSCs on collagen coated plates. The IFT88^−/−^ MuSCs did not differ from ciliated controls in their spontaneous differentiation and exhibited a comparable capacity to form MyHC positive myotubes (Supplementary Fig. 2e), consistent with our hypothesis that primary cilia mediate MuSC proliferation and expansion.

To determine if primary cilia are required for MuSC self-renewal in vivo, we performed engraftment studies using control and IFT88^−/−^ MuSCs transduced with a GFP/luciferase expression vector, which enables assessment of the dynamics of MuSC engraftment and contribution to regeneration over time by non-invasive bioluminescence imaging (BLI). Immunodeficient Nod Scid Gamma (NSG) mice were irradiated to deplete competing endogenous MuSCs. Equal numbers of GFP/luc expressing control and IFT88^−/−^ MuSCs were then injected into the *Tibialis anterior* (TA) muscles and engraftment potential measured over time using BLI, as previously described^6,32,33^ (Fig. 2e). We found that IFT88^−/−^ MuSC engraftment was significantly reduced compared to controls (Fig. 2f and Supplementary Fig. 2f). These data show that cilia are crucial to the self-renewal and expansion capacity of transplanted MuSCs in vivo.

### Smoothened agonist (SAG) promotes MuSC expansion in vivo

**Fig. 3:**
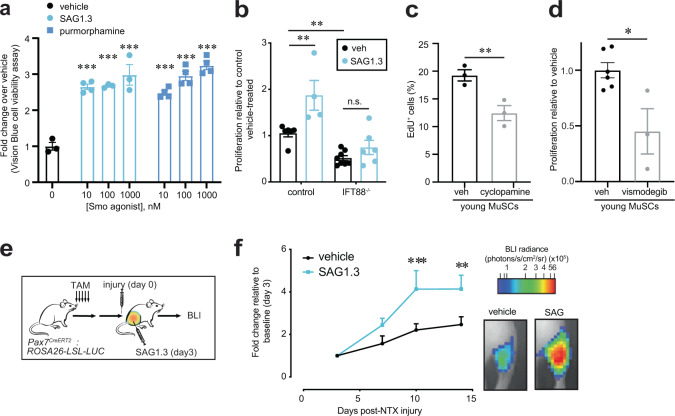
SMO agonist treatment promotes MuSC expansion. **a** SMO agonists SAG1.3 and purmophamine promote MuSC proliferation in a dose-dependent manner (*n* = 3–4 biological replicates per condition, isolated from independent mice and each performed in 3 technical replicates). **b** Proliferation of control or IFT88^−/−^ MuSCs treated with vehicle (veh) or SMO agonist SAG1.3 (50 nM), shown as fold change normalized to control vehicle treated (*n* = 5 control veh, 4 control SAG1.3, 8 IFT88^−/−^ veh, 6 IFT88^−/−^ SAG1.3). **c** Percentage of EdU^+^ vehicle or 5 µM cyclopamine (SMO antagonist) treated MuSCs (*n* = 3 mice per condition). **d** Vismodegib (100 nM, SMO antagonist) inhibits MuSC proliferation as determined by Vision Blue assay (*n* = 7 veh and 3 vismodegib treated). **e**, **f** Expansion of endogenous MuSCs in Pax7CreERT2;Rosa26-LSL-Luc mice treated with tamoxifen (TAM) to label resting MuSCs and assayed by BLI post-notexin injury. **e** Experimental scheme. **f** Left: BLI (*n* = 8 mice per condition, individual data points in Supplementary Fig. 3a). Right: Representative BLI image. *P < 0.05, **P < 0.01, ***P < 0.001. Mann–Whitney test (**c**, **d**). ANOVA test with Fisher’s LSD for multiple comparisons (**a**, **b**). Significance relative to vehicle (0 nM) (**a**); 2 way ANOVA test with Fisher’s LSD for multiple comparisons per time point (**f**). Source data are provided as a Source Data file. Means+s.e.m.

Primary cilia have an essential role in mediating Hh signaling in vertebrates^34^. The Hh receptor Patched (PTCH1) and the downstream GPCR Smoothened (SMO) are highly enriched in the primary cilium and regulate Hh signaling via downstream GLI transcription factors that have been shown to play a role in proliferation and renewal of diverse stem cell types^34,35^. To test if Hh signaling plays a role in MuSC self-renewal and expansion, we used small-molecule SMO agonists (SAG1.3 and Purmorphamine) to activate Hh signaling^36^. We found SAG1.3 and Purmorphamine stimulated MuSC expansion in a dose-dependent manner (Fig. 3a). Additionally, we found that treatment of control, but not IFT88^−/−^ MuSCs, with the SMO agonist SAG1.3 led to a robust increase in proliferation (Fig. 3b). Thus, activation of the Hh signaling pathway promotes cilia-dependent MuSC expansion. Similarly, inhibition of Hh signaling using SMO antagonists cyclopamine or vismodegib^36,37^ markedly reduced proliferation, as assessed by EdU incorporation (Fig. 3c) or VisionBlue, a Resazurin-based dye that becomes fluorescent upon reduction by metabolically active cells (Fig. 3d). To further study if stimulation of the Hh pathway promotes muscle regeneration in vivo, we used a transgenic mouse model we previously developed, Pax7^CreERT2^; Rosa26-LSL-Luc to monitor the dynamics of endogenous MuSC expansion over a 2-week time course using BLI^32^. We found that a single intramuscular injection of SAG1.3 significantly increased endogenous MuSC expansion at days 10 and 14 post-injury compared to vehicle treated (Fig. 3e, f and Supplementary Fig. 3). These results demonstrate that ciliary Hh signaling via SMO is critical for the proliferative capacity of MuSCs, and that stimulating SMO can lead to increased MuSC expansion during regeneration.

### Aged MuSCs exhibit decreased ciliation and expression of Hh downstream target Gli2

**Fig. 4:**
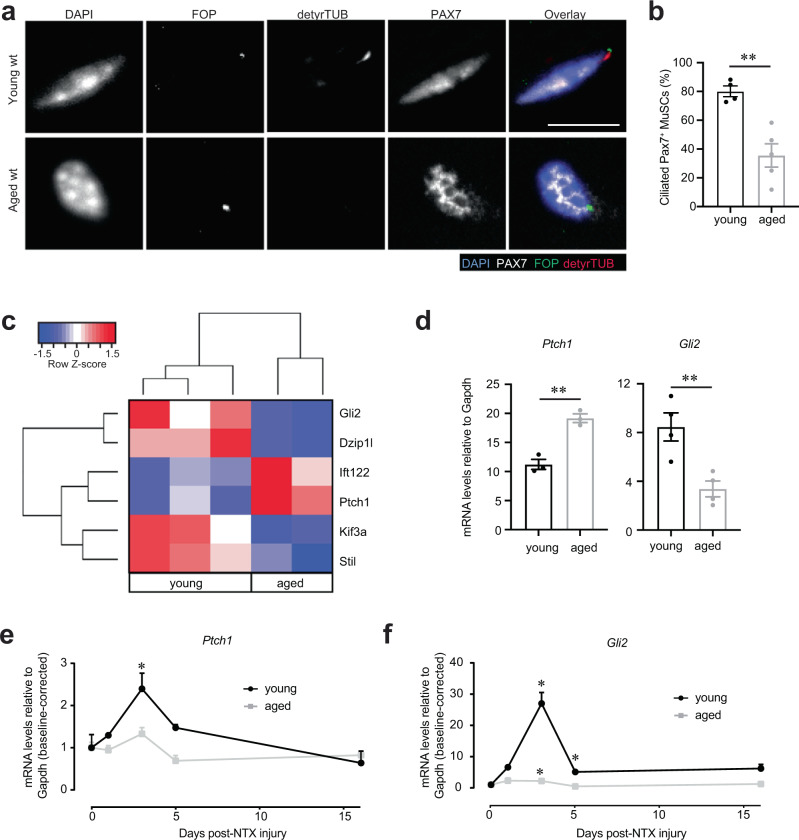
Aged MuSCs present decreased ciliation and Hedgehog signaling. **a** Representative confocal images of uninjured/resting EDL myofibers of young (2 months) and aged (>24 months) C57BL/6 wild-type (wt) mice showing cilia immunostaining in Pax7^+^ MuSCs. Scale bars: 10 μm. DAPI, blue; PAX7, white; FOP, green; detyrosinated tubulin, red. **b** Percent of ciliated Pax7+ MuSCs quantified from isolated myofibers (*n* = 58 myofibers total per age group isolated from *n* = 4 young and *n* = 5 aged mice, average percent of ciliated MuSCs per mouse is shown). **c** Heat map of differentially expressed Hh pathway genes in young and aged MuSCs. **d** Expression of the negative regulator of Hh signaling, *Ptch1*, is increased in aged MuSCs, while the target gene *Gli2* is decreased in aged MuSCs compared to young as determined by qPCR analysis (*n* = 4 mice per age group). **e**, **f** Expression of *Ptch1* (**e**) and *Gli2* transcription factor (**f**) after *Tibialis anterior* (TA) muscle injury (notexin) (*n* = 3–7 mice per timepoint and age group, individual data points in Supplementary Fig. 4c, d). *P < 0.05, **P < 0.01, ***P < 0.001 ****P < 0.0001. Mann–Whitney test (**b**, **d**). ANOVA test for group comparisons and significant difference for each timepoint relative to Day 0 by Fisher’s LSD test (**e, f**). Source data are provided as a Source Data file. Means+s.e.m.

Since MuSC function declines with aging and ablation of cilia leads to a loss of young MuSC self-renewal capacity, we hypothesized that aged MuSCs harbor a ciliation defect that contributes to their loss of stemness and regenerative capacity. We therefore assessed the ciliation status of MuSCs during aging. We compared the cilia of young and aged MuSCs present on isolated myofibers. By immunofluorescence analysis, we identified the cilia of PAX7-positive MuSCs using antibodies directed against detyrosinated tubulin and the centriolar marker FOP (Fig. 4a and Supplementary Fig. 4a). Aged MuSCs had a significant decrease in the number of both long and short cilia compared to young, with only approximately 30% of aged MuSCs remaining ciliated (Fig. 4b and Supplementary Fig. 4b). Upon analysis of transcriptome data from young and aged MuSCs, we identified several differentially expressed ciliary genes (i.e. *Ift122, Kif3a, Dzip1l, Stil*) (Fig. 4c). Components of the Hh signaling pathway (i.e. *Ptch1, Gli2*), known to signal through the primary cilium^16^, were among the top hits (Fig. 4c). Moreover, expression of Hh signaling genes, the negative regulator, *Ptch1*, and the downstream effector, *Gli2*, were dysregulated in aged compared to young MuSCs (Fig. 4d), not only at a basal state, but also in muscles post-injury (Fig. 4e, f and Supplementary Fig. 4c, d). In an injury time course, increased *Ptch1* expression was observed at the uninjured and at 1, 3 and 16 days post-injury in aged compared to young mice, while *Ptch1* expression was transiently induced in young mice only in response to injury (Fig. 4e, Supplementary Fig. 4c). *Gli2*, which is transiently upregulated at day 3 post-injury in young, exhibits decreased expression in aged compared to young at this timepoint (Fig. 4f, Supplementary Fig. 4d). These data demonstrate that aged MuSCs have a cell-intrinsic ciliation defect, which likely results in a blunted response to Hh signaling in aged MuSCs.

### SMO agonist (SAG1.3) promotes young and aged MuSC proliferation and function

**Fig. 5:**
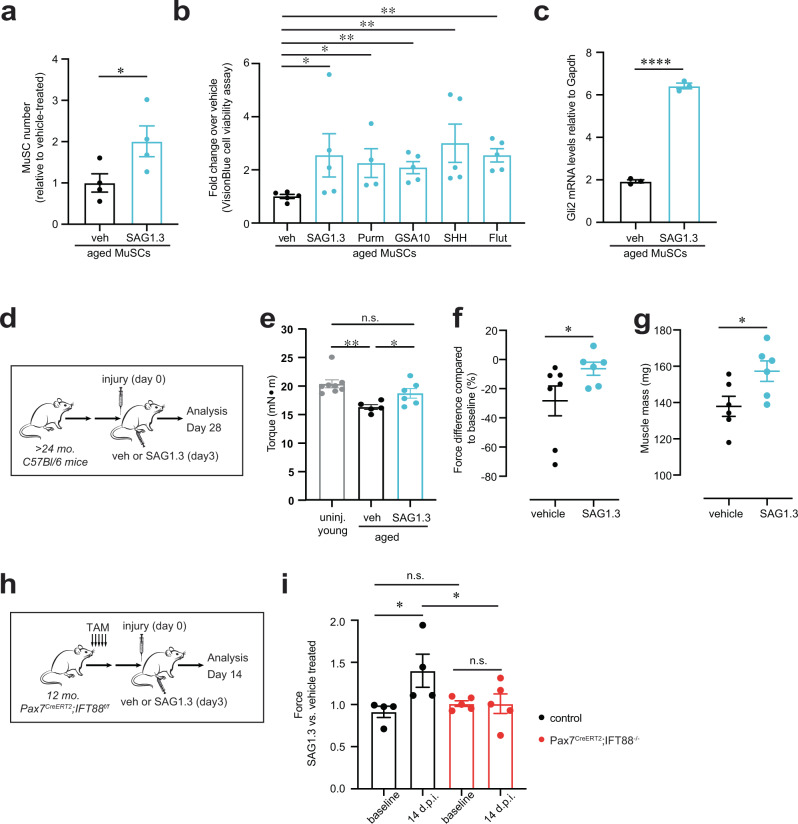
SMO agonist promotes MuSC proliferation and function in aged MuSCs. **a** Proliferation of aged MuSCs (>24 mo) treated with vehicle (veh) or SAG1.3 (50 nM), shown as fold change normalized to vehicle treated (*n* = 4 mice per condition, each with 3 technical replicates). **b** Proliferation of aged MuSCs treated with SMO agonist, shown as fold change normalized to vehicle treated (50 nM SAG1.3; 1 µM Purm, Purmorphamine; 0.1 µg/ml SHH, Sonic-Hedgehog; 1 µM GSA-10; 25 nM; Flut, Fluticasone) (*n* = 5 technical replicates per condition). **c** Expression levels of SMO downstream target *Gli2* in MuSCs 24 h after vehicle or SAG treatment (*n* = 3 mice per condition). **d**–**g** Aged (>24 months) mice were injected with vehicle or SAG post-notexin injury and muscle regeneration was analyzed 4 weeks later as force recovery. **d** Experimental scheme. **e** Plantar flexion tetanic torque (absolute values) (*n* = 6 mice per condition). **f** Plantar flexion tetanic torque relative to baseline (*n* = 6 mice per condition). (**g**) *Gastrocnemius* muscle mass (*n* = 6 mice per condition). **h**, **i)** Aged control Pax7^CreERT2^;IFT88^f/f^ (control, corn oil) and Pax7^CreERT2^;IFT88^−/−^ (IFT88^−/−^, tamoxifen) mice treated with vehicle or SAG1.3 post-notexin injury and muscle regeneration was analyzed 14 days later. **h** Experimental scheme. **i** Force measurement was normalized to contralateral leg injected with vehicle. Normalized force is shown at baseline (prior to treatment) and 14 days post-injury (d.p.i) (*n* = 4 control and *n* = 5 IFT88^−/−^ mice). *P < 0.05, **P < 0.01, ***P < 0.001 ****P < 0.0001. Paired *t* test compared to vehicle treated (**a**, **b)**. Mann–Whitney test (**c**, **f**, **g**). ANOVA test with Fisher’s LSD for multiple comparisons **(e**, **i)**. Source data are provided as a Source Data file. Means + s.e.m n.s., non-significant. uninj., uninjured.

Aged MuSCs are known to have reduced proliferative capacity, which correlates with reduced regeneration of muscle post-injury. To assess if Hh stimulation in the remaining ~30% of ciliated aged MuSCs suffices to improve muscle regeneration in the aged, we treated isolated MuSCs from aged mice with SAG1.3. This resulted in a significant increase in proliferation (Fig. 5a). To confirm this finding, we treated aged MuSCs with other known agonists of SMO, including small molecules (purmorphamine and GSA-10)^36^, the glucocorticoid fluticasone^38^, and the Sonic-Hedgehog (Shh) ligand which activates signaling by binding PTCH1 and derepressing SMO^39^. We found that each of the SMO agonists we tested significantly enhanced aged MuSC proliferation (Fig. 5b). Moreover, we validated that SAG1.3 treatment of aged MuSCs increased expression of the downstream Hh target *Gli2* (Fig. 5c).

To determine if SAG1.3 injection could enhance the function of endogenous aged MuSCs juxtaposed to myofibers in muscle tissue, we injected SAG1.3 into the *Gastrocnemius* (GA) muscles post-injury. Notably, we observed a significant increase in the absolute strength, strength of each mouse relative to its baseline strength, and muscle mass of SAG1.3-treated aged muscles post-injury (Fig. 5d–g). Remarkably, the absolute strength of SAG1.3-treated aged muscles post-injury was similar to that of uninjured, young muscle (Fig. 5e). Since other ciliated cells such as fibroadipogenic progenitors (FAPs) are known to play a role in muscle regeneration^40–42^, we tested the specificity of SAG1.3 treatment for MuSCs in vivo post-injury. We injected the GA muscles of Pax7^CreERT2^; IFT88^f/f^ (control) or Pax7^CreERT2^; IFT88^−/−^ mice with SAG1.3 or vehicle post-injury (Fig. 5h). We found SAG1.3 increased strength only in control mice, but not in Pax7^CreERT2^;IFT88^−/−^ in which cilia were specifically ablated on MuSCs, confirming that SAG1.3 enhances muscle regeneration in vivo by promoting MuSC proliferation in a cilia-dependent manner (Fig. 5i). These studies show that pharmacological stimulation of ciliary Hedgehog signaling in aged MuSCs significantly enhances recovery of muscle mass and strength post-injury.

## Discussion

**Fig. 6:**
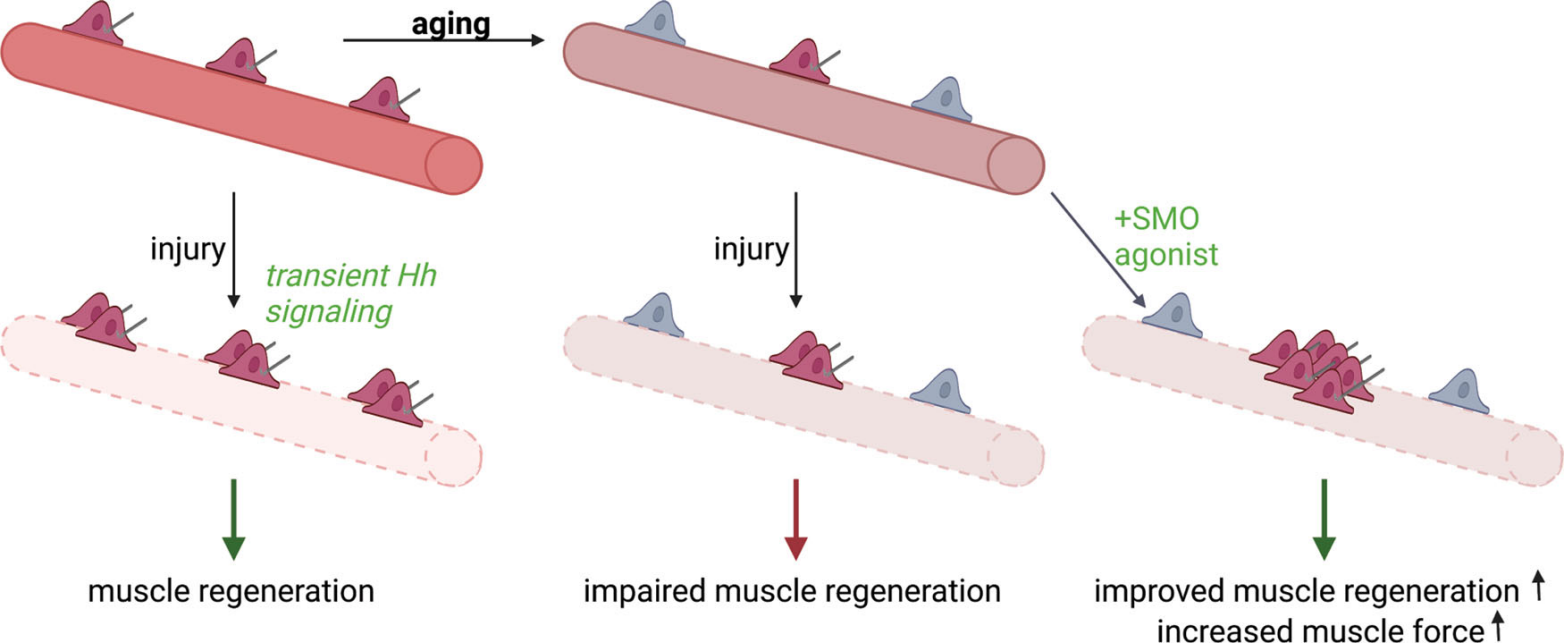
Scheme of primary cilia function in muscle regeneration. Notexin-induced muscle injury induces transient Hedgehog signaling in MuSCs, which results in MuSC expansion, a critical step in muscle regeneration. The primary cilium is lost in a significant fraction of aged MuSCs, and exogenous activation of Hedgehog signaling increases muscle force in aged. Created with BioRender.com.

Primary cilia are cellular protrusions found on most mammalian cells that serve as sensory organelles that detect diverse signals such as light, growth factors, and morphogens^43^. Perturbation of ciliary proteins in all ciliated cell types leads to a broad range of genetic disorders known as “ciliopathies”, which can affect numerous cell types and tissues. Hypotonia and muscle flaccidity is one common clinical manifestation, for example in Joubert Syndrome. This is at least in part a consequence of disrupted motor coordination^44^. Here, we uncover a distinct role for primary cilia on MuSCs in mediating the Hedgehog signaling pathway to promote muscle regeneration in vivo (Fig. 6). Additionally, we provide novel evidence of the profound role of this pathway in muscle regenerative function in aging.

There is a paucity of information regarding the significance of ciliation in age-related diseases. Here, we demonstrate that an absence of ciliation is a cell-intrinsic change in aged MuSCs that diminishes their function. We propose that loss of cilia contributes to a reduction in muscle regenerative capacity with aging. We note that the age-dependent loss in ciliation is not complete, but instead resembles the effect on MuSC ciliation of heterozygous deletion of IFT88 in young adult mice. MuSC primary cilia appear to be highly sensitive to changes in levels of ciliary protein expression and thus particularly susceptible to aging-related changes. We show that the decrease in ciliation in aged MuSCs results in impairment of Hh signaling, suggesting an important role for Hh in muscle regeneration. Although there is limited in vivo evidence for the role of Hh signaling in muscle regeneration, previous studies have shown that SMO inhibition by cyclopamine treatment of injured muscles results in muscle fibrosis and increased inflammation^45^. Additionally, in the context of aging, transduction of a *Shh*-expressing vector was shown to boost muscle repair to levels comparable to those found in much younger mice^46^. Smoothened has been targeted for different therapeutic indications, including agonists (SAG, GSA-10) and antagonists for the treatment of basal cell carcinoma^36^. Here, we provide a link between Hh signaling and ciliation in MuSCs and show that Hh signaling is important for the regenerative capacity of muscle in vivo. Furthermore, treatment with a small molecule that restores Hh signaling in aged MuSCs improves endogenous stem cell function and significantly augments muscle regenerative capacity after injury of aged muscles (Fig. 6). Although activation of Hh signaling can increase tumorigenesis (including medulloblastoma and basal cell carcinomas)^47^, these undesired effects may be circumvented by the transient, localized nature of the SMO agonist treatment which we show can robustly enhance MuSC expansion leading to increased muscle strength after injury in aged mice.

Here, we demonstrate that disruption of cilia is an intrinsic change in aged MuSCs that impedes their function in regeneration. In the absence of ciliary Hh signaling, the repair of muscle damage by endogenous or transplanted MuSCs is hindered, and strength is not restored after injury. The aged niche and extrinsic factors within the microenvironment may also play a role, and experiments designed to determine the cell source and identity of the endogenous Hh signals in muscle after injury are clearly warranted. Desert Hh (DHH) has been reported to be expressed by Schwann cells post-cardiotoxin injury^41^. Since loss of neuromuscular junctions (NMJ) plays a prominent role in the decline in muscle function with aging^48^, it is possible that impairment of Schwann cells contributes to a loss of Hh signaling and the observed decline in MuSC function with aging. Further, we show that there are notable differences in how *Ptch1* and *Gli2* expression changes with aging, such that the basal (uninjured) levels of *Ptch1* are increased in aged MuSCs, while *Gli2* expression is decreased. There are a number of potential explanations for this divergent response, including de-repression of Hh signaling in the absence of ciliation and the presence of *Ptch1* activators that are non-responsive to Hh^49^. Regardless of these differences in basal *Ptch1* and *Gli2* expression in aged MuSCs, the Hh signaling response is dramatically blunted in aged muscle in response to injury.

Mesenchymal stem and progenitor cells are broadly ciliated and play an important role in maintaining stem cell fate by regulating lineage specification, proliferation, and differentiation^50–52^. For example, in adipose tissue, preadipocytes are ciliated and their ablation leads to severe defects in white adipose tissue expansion^25,53^. Cilia also play a critical role in cartilage and bone development^54^. In skeletal muscle, although the function of cilia on MuSCs was not previously discerned in vivo, the presence of cilia was shown in another progenitor population, fibroadipogenic progenitors, and shown to play an important role in regulating its adipogenic fate in the context of injury and in a model of Duchenne Muscular Dystrophy^41^. Thus, ciliation of multiple progenitor cell populations may be critical to tissue homeostasis and function.

Many signaling pathways are known to be mediated by the primary cilium. Here, we implicate one of the well-studied functions of primary cilia, transduction of Hh signaling, in muscle stem cell function. Additional ciliary signaling pathways may also play a role in muscle regeneration. These include pathways activated in response to inflammation, since inflammatory signaling plays a critical role in almost all stages of muscle regeneration^32,55,56^ and is known to be transduced by the primary cilium in other cell contexts^57,58^. Additionally, since primary cilia are sensory organelles, they could sense chemotactic cues and direct migration of MuSCs to sites of injury to promote regeneration as seen with other mesenchymal stem cells^59^. Upon activation, MuSCs are known to migrate to regenerate injured areas^60^, and cilia may well play an essential role in sensing key chemotactic cues. Our observation that loss of ciliation in MuSCs results in a severe dysregulation of calcium and G protein-coupled signaling is consistent with the MuSC primary cilium mediating a multitude of signaling pathways. Indeed, how the primary cilium senses and integrates multiple signaling pathways into ciliary effector proteins to coordinate the initiation of myogenesis during development remains an intriguing question^12^. Notably, ciliogenesis has been linked to planar cell polarity non-canonical Wnt signaling^61^. Since Wnt signaling in MuSCs has been shown to control quiescence via the planar cell polarity pathway^62^ and its dysregulation in aging muscles alters MuSC function^10^, loss of primary cilia may well underlie the loss of non-canonical Wnt signaling. Our data underscore the profound functional importance of the primary cilium in Hedgehog signaling in MuSCs during the regeneration of damaged tissue. These findings provide fresh insights into the etiology of signaling dysfunction in aging and a potential therapeutic strategy to augment aged muscle regeneration.

## Methods

### Mice

All experiments and protocols were performed in compliance with the institutional guidelines of Stanford University and Administrative Panel on Laboratory Animal Care (APLAC). The laboratory animal care program at Stanford University is accredited by the Association for the Assessment and Accreditation of Laboratory Animal Care (AAALAC International). Animals were group housed on a 12-h:12-h light:dark cycle and regulated temperature (68–79 °F) and humidity levels (30–70%) in the Stanford University’s Veterinary Service Center (VSC) and fed with food and water ad libitum. All studies were conducted in accordance with the GSK Policy on the Care, Welfare and Treatment of Laboratory Animals and were reviewed the Institutional Animal Care and Use Committee either at GSK or by the ethical review process at the institution where the work was performed. Aged (>24 mo.) and young (2–4 mo.) C57BL/6 mice were obtained from Jackson Laboratory. Mouse transgenic strains were purchased from Jackson Laboratory (NOD-SCID No. 005557; *Pax7*^*CreERT2*^ No. 017763; *IFT88*^*flox/flox*^ *No*. 022409; *Rosa26-LSL-Luc* No. 005125; *IFT88*^*flox/flox*^ No. 022409; and *Centrin2-eGFP; Arl13b-mCherry* No. 027967^24^). Double-transgenic *Pax7*^*CreERT2*^*;Rosa26-LSL-Luc* were generated as described previously^32^. Double-transgenic *Pax7*^*CreERT2*^*;IFT88*^*flox/flox*^ (*IFT88*^*f/f*^) were generated by crossing *Pax7*^*CreERT2*^ mice obtained from Jackson Laboratory^63^ and *IFT88*^*f/f*^ obtained from Jackson Laboratory^26^. For *Pax7*^*CreERT2*^*;Rosa26-LSL-Luc* and *Pax7*^*CreERT2*^*;IFT88*^*flox/flox*^ (*IFT88*^*f/f*^) mice experiments, we treated 8-week-old or 12-month-old male mice with five consecutive daily intraperitoneal injections of tamoxifen (50 mg/kg; Sigma Aldrich, catalog # 10540-29-1) and performed intramuscular notexin injury one week after the last tamoxifen injection. We validated these genotypes by appropriate PCR-based strategies.

### Muscle stem cell isolation

We isolated and enriched muscle stem cells as previously described (Supplementary Fig. 5)^6,31–33^. Briefly, hindlimb muscles were minced and digested using a collagenase type 2 (Worthington Biochemical, catalog # LS004177) and dispase II (Thermo Fisher Scientific, catalog # 17105041) solution by the gentleMACs Octo Dissociator (Miltenyi). Subsequently, single cells were depleted for hematopoietic lineage expressing and non-muscle cells (CD45^−^/CD11b^−^/Sca1^−^/CD31^−^) (Biotin anti-CD45, BD Biosciences catalog # 553078, clone 30F11, 1:500 dilution; Biotin anti-CD11b, BD Biosciences, catalog # 553309, clone M1/70, 1:200 dilution, Biotin anti-Sca1 (Ly-6A/E), BD Biosciences catalog # 553334, clone E13-161.7, 1:200 dilution and Biotin anti-CD31, eBioscience catalog # 13-0311-82, clone 390, 1:200 dilution) using a magnetic bead column (Streptavidin MicroBeads, Miltenyi Biotec, catalog # 130-048-101). The remaining Lin^-^ cell mixture was then subjected to FACS analysis to sort for MuSCs co-expressing CD34 (Anti-Mouse CD34 eFluor 660, clone RAM34, 1:67 dilution, eBioscience, catalog # 50-0341-82) and Integrin-α7 (Anti-Integrin alpha 7 antibody conjugated to phycoerythrin (PE), clone R2F2, 1:200 dilution, Ablab, catalog # 10ST215) markers and negative for Lin (Streptavidin-APC-Cy7, 1:200 dilution, BD Biosciences, catalog # 554063). We generated and analyzed flow cytometry scatter plots using FlowJo v10.0.

### Muscle stem cell transplantation

We co-injected 250 GFP/luc IFT88^−/−^ or control MuSCs into the *tibialis anterior* (TA) muscles of recipient NOD-SCID mice as previously described^6,31–33^. GFP/luc IFT88^−/−^ or control MuSCs were obtained by isolating MuSCs from IFT88^−/−^ or control mice (2–4 mo.) post-tamoxifen injection and transducing with a luc-IRES-GFP lentivirus (GFP/luc virus) on day 1 of culture for a period of 24 hr before transplantation (see below “Cell culture” section for details). We compared cells from different conditions by transplantation into the TA muscles of contralateral legs in the same mice. Four weeks after transplantation, mice were euthanized, and the TAs were collected for analysis.

### Bioluminescence imaging

We performed bioluminescence imaging (BLI) using a IVIS Spectrum system (Perkin Elmer), as previously described^6,31–33^. Briefly, we anesthetized mice using isofluorane inhalation and administered 120 μL D-luciferin (0.1 mmol kg^−1^, reconstituted in PBS; Caliper LifeSciences) by intraperitoneal injection. We acquired BLI using a 60 s exposure at F-stop=1.0 at 5 min after luciferin injection. Digital images were recorded and analyzed using Living Image software (Perkin Elmer). We analyzed images with a consistent region-of-interest (ROI) placed over each hindlimb to calculate a bioluminescence signal. We calculated a bioluminescence signal in radiance (p s^−1^ cm^−2^ sr^−1^) value of 10^4^ to define an engraftment threshold. This radiance threshold of 10^4^ is approximately equivalent to the total flux threshold in p/s reported previously. This BLI threshold corresponds to the histological detection of one or more GFP + myofibers^6,31–33^. We performed BLI imaging every week after transplantation.

### Muscle injury

We used an injury model entailing intramuscular injection of 20 or 40 μl of notexin (10 μg ml^−1^; Latoxan, catalog# L8104) into the TA muscle or GA muscle respectively. Notexin is a phospholipase A2 neurotoxin peptide extracted from snake venom that acts as a myotoxin upon intramuscular injection. It promotes myofiber necrosis which promotes muscle stem cell activation and proliferation to regenerate the injured muscle^64^. When indicated, 3 days after injury SAG (175 µg/kg, Tocris, catalog # 4366) or vehicle control (PBS) was injected into the GA muscle as previously described^41^. We collected tissues at times indicated for analysis.

For *Pax7*^*CreERT2*^*; Rosa26-LSL-Luc* mice experiments, we treated mice with five consecutive daily intraperitoneal injections of tamoxifen to activate luciferase expression under the control of the *Pax7* promoter. A week after the last tamoxifen injection, mice were subjected to intramuscular injection of notexin, which we designated as day 0 of the assay. Three days later either SAG (175 µg/kg, Tocris, catalog # 4366) or vehicle control (PBS) was injected into the TA or GA muscle. Bioluminescence was assayed at days 3, 7, 10, and 14 post-injury.

### Immunofluorescence staining and imaging

We collected and prepared recipient TA muscle tissues for histology as previously described^32^. We fixed transverse sections from muscles using 4% PFA, blocked and permeabilized using PBS/1% BSA/0.1% Triton X-100 and incubated with anti-LAMININ (Millipore, clone A5, catalog # 05-206, 1:200) and then with AlexaFluor secondary Antibodies (Jackson ImmunoResearch Laboratories, 1:200) or wheat germ agglutinin-Alexa 647 conjugate (WGA, Thermo Fisher Scientific). We counterstained nuclei with DAPI (Invitrogen).

For myofibers and MuSCs, we performed fixation using 4% PFA, blocking and permeabilization using PBS/1% BSA/0.1% Triton X-100 and staining with primary antibodies anti-detyrosinated tubulin (abcam, catalog # ab48389, 1:100), anti-IFT88 (ProteinTech, catalog # 13967-1-ap, 1:500), anti-PAX7 (Santa Cruz Biotechnology, catalog # sc-81648, 1:50), anti-FOP (Abnova, catalog #H00011116-M01, clone 2B1, 1:1000), anti-MyHC (clone MF20, Thermo Fisher Scientific, catalog # 14-6503-82, 1:500) and then with AlexaFluor secondary Antibodies (Jackson ImmunoResearch Laboratories, 1:500). We counterstained nuclei with DAPI (Invitrogen).

Confocal images of myofibers were acquired on a Marianas spinning disk confocal (SDC) microscopy (Intelligent Imaging Innovations) with a ×40/0.9 N.A. objective to capture multiple consecutive focal planes. Muscle transverse sections images were acquired on a KEYENCE BZ-X700 all-in-one fluorescence microscope (Keyence) with ×20/0.75 N.A. objectives. We analyzed the myofiber cross-sectional area using the Keyence Software that identified the fibers and segmented the fibers in the image to analyze the area of each fiber. For fiber area at least 10 fields of LAMININ-stained myofiber cross-sections encompassing over 400 myofibers were captured for each mouse. For percent ciliation and cilia length, we analyzed young and aged MuSCs on at least 30 myofibers isolated from 5 independent mice using Intelligent Imaging Innovations software and the measurement tool. Data analyses were blinded. The researchers performing the imaging acquisition and scoring were unaware of conditions given to sample groups analyzed.

### Hydrogel fabrication

We used polyethylene glycol (PEG) hydrogels from PEG precursors, synthesized as described previously^31^. Briefly, we produced 12-kPa (Young’s modulus) hydrogels in 1 mm thickness functionalized with laminin to cover the surface area of 12-well or 24-well culture plates.

### Myofiber isolation and culture

EDL myofibers were isolated as previously described^65^. Briefly, the extensor digitorum longus (EDL) muscle was dissected and digested in 0.2% Collagenase type B (catalog # 11088831001, Roche) in DMEM at 37 °C for 1 h. Single myofibers were isolated by triturating the digested EDL muscle with polished Pasteur pipettes and then fixed with PFA 4%.

### Cell culture

Following FACs isolation, we resuspended MuSCs in myogenic cell culture medium containing DMEM/F10 (50:50), 15% FBS, 2.5 ng ml^−1^ fibroblast growth factor-2 and 1% penicillin-streptomycin. We added the indicated doses (see respective Figure legends) 50 nM of SAG1.3 (Cayman Chemical Company, catalog # 11914), 1 µM of GSA-10 (Sigma-Aldrich, catalog # SML1171), 25 nM of Fluticasone (Sigma-Aldrich, catalog # F9428), 5 µM of Cyclopamine (STEMCELL Technologies, catalog #72072), 1 µM Purmorphamine (Selleck Chemical, catalog # S3042), 100 nM of Vismodegib (Selleck Chemicals, catalog # S1082) or 0.1 µg/ml SHH (R&D Systems, catalog # 461-SH-025) to MuSCs cultured on collagen coated dishes for the first 24 h. The cells were then trypsinized and cells reseeded onto hydrogels for an additional 6 days of culture. All treatments were compared to their solvent (DMSO) vehicle control.

For IFT88^−/−^ MuSCs transplant studies, we infected MuSCs with lentivirus encoding elongation factor-1α promoter-driven luc-IRES-GFP (GFP/luc virus) for 24 h in culture as described previously^6^. Cells were assayed for GFP 48 h post-infection using an inverted fluorescence microscope (Carl Zeiss Microimaging).

### Proliferation assays

To assay proliferation, we seeded MuSCs on flat hydrogels at a density of 500 cells per cm^2^ surface area. We counted cell number using a hemocytometer. We collected cells at indicated timepoints by incubation with 0.5% trypsin in PBS for 5 min at 37 °C and quantified them using a hemocytometer at least 3 times. Additionally, we used the VisionBlue Quick Cell Viability Fluorometric Assay Kit (BioVision, catalog # K303) as a readout for cell growth in culture. Briefly, we incubated MuSCs with 10% VisionBlue in culture medium for 4 h, and measured fluorescence intensity on a fluorescence plate reader (Infinite M1000 PRO, Tecan) at Ex= 530–570 nm, Em=590-620 nm. Data analyses were blinded, where researchers performing cell scoring were unaware of the treatment condition given to sample groups analyzed.

### Dose curve on MuSC proliferation

To assess MuSC proliferation with different drug doses, we seeded 400 MuSCs isolated from young mice (2–4 mo.) on collagen coated 96 well plates in myogenic cell culture medium containing DMEM/F10 (50:50), 15% FBS, 2.5 ng ml^−1^ fibroblast growth factor-2 and 1% penicillin-streptomycin. We performed 3-5 replicates per condition plated with 3 doses (1, 100 or 1000 nM). Each plate contained vehicle controls. Small molecules were added at a 2X concentration to achieve the final concentration. To avoid cell-washout effects, we did not change the medium throughout the assay. Proliferation was assayed at 7 days post-plating by using the VisionBlue Quick Cell Viability Fluorometric Assay Kit (BioVision, catalog # K303) as a readout for cell growth in culture. Briefly, we incubated live MuSCs with 10% VisionBlue in culture medium for 4 h, and measured fluorescence intensity on a fluorescence plate reader (Infinite M1000 PRO, Tecan) at Ex= 530–570 nm, Em=590-620 nm. Proliferation was normalized to the average value of vehicle treated for each plate. Data analyses were blinded, where researchers performing cell scoring were unaware of the treatment condition given to sample groups analyzed.

### Quantitative RT-PCR

We isolated RNA from MuSCs using the RNeasy Micro Kit (Qiagen). We reverse-transcribed cDNA from total mRNA from each sample using the SensiFAST™ cDNA Synthesis Kit (Bioline). We subjected cDNA to RT-PCR using TaqMan Assays (Applied Biosystems) or SYBR Greem PCR Master Mix (Applied Biosystems) in an ABI 7900HT Real-Time PCR System (Applied Biosystems). We cycled samples at 95 °C for 10 min and then 40 cycles at 95 °C for 15 s and 60 °C for 1 min. To quantify relative transcript levels, we used 2 − ΔΔCt to compare experimental and control samples and expressed the results relative to *Gapdh*. Raw Ct values for qPCRs are provided in the Source Data file.

TaqMan Assays (Applied Biosystems) were used to quantify *Gli2 (*Thermo Fisher Scientific, Catalog # Mm01293117_m1), *Ptch1* (Thermo Fisher Scientific, Catalog # Mm00436026_m1), *Cep164* (Thermo Fisher Scientific, Catalog # Mm00553106_m1), *Kif3a* (Thermo Fisher Scientific, Catalog # Mm01288585_m1), *Gapdh (*Thermo Fisher Scientific, Catalog # Mm01162710_m1) in samples according to the manufacturer instructions with the TaqMan Universal PCR Master Mix reagent kit (Applied Biosystems). For Taqman qPCR, multiplex qPCR enabled target signals (FAM) to be normalized individually by their internal *Gapdh* signals (VIC).

We analyzed *Mcm9*, *Cdk13* using SYBR Green qPCR. For SYBR Green qPCR we used the following primer sequences: *Mcm9*, forward 5′-CAAGCATCCATGAAGCAATG-3′, reverse 5′-GATGGTGGTCCTTGTGTTCAG-3′; *Cdk13*, forward 5′-AGACGTGGAACCCTCCAAA-3′, reverse 5′-TCATCAGTCATGCCCATCTG-3′; *Gapdh*, forward 5′-TTCACCACCATGGAGAAGGC-3′, reverse 5′-CCCTTTTGGCTCCACCCT-3′.

### Flow cytometry

We assayed EdU as a readout of proliferation for MuSCs after 7 days in culture on hydrogels, after an initial acute (24 hr) treatment of vehicle (DMSO), SAG, or cyclopamine, or of control and IFT88^−/−^ MuSCs. EdU incorporation was assessed using the Click-iT™ EdU Pacific Blue™ Flow Cytometry Assay Kit (Thermo Fisher Scientific, catalog #C10418) according to the manufacturer’s recommendation. Briefly, we incubated MuSCs for 1 h with EdU for a final concentration of 20 μM after 7 days of proliferation on hydrogels. Cells were trypsinized and fixed in 4% PFA/PBS for 15 min at room temperature, followed by 2 rinses in 1%BSA/PBS. Cells were permeabilized in cold methanol for 10 min. Cells were kept in 1%BSA/PBS for 30 min at room temperature, followed by 2 rinses in 1%BSA/PBS. Samples were then incubated in a freshly made Click-iT reaction cocktail for 30 min at room temperature, followed by 1 rinse in 3%BSA/PBS and 1 rinse in PBS. Samples were then incubated in 7AAD in PBS for 30 min at room temperature followed by 1 rinse in PBS. We analyzed the cells on a FACS LSR II cytometer using FACSDiva software (BD Biosciences). Analysis of EdU positive cells was performed using FlowJo Software (BD Biosciences).

### Knockdown using siRNA

Electroporation was performed using a Neon Transfection System (Invitrogen) with ON-TARGETplus pool siRNAs against Cep164 (Cat No. L-057068-01-0005, Dharmacon), Kif3a (Cat No. L-042111-01-0005, Dharmacon) or control non-targeting siRNAs (Cat No. D-001810, Dharmacon) to MuSCs 24 h after isolation. After electroporation, MuSCs were plated and proliferation was assessed 7 days later using a VisionBlue viability fluorescence readout (see *Proliferation assays* section).

### In vivo muscle force measurement

The peak isometric torque (N•mm) of the ankle plantarflexors was assessed as previously described^66,67^. Briefly, the foot of anesthetized mice was placed on a footplate attached to a servomotor (model 300C-LR; Aurora Scientific). Two Pt-Ir electrode needles (Aurora Scientific) were inserted percutaneously over the tibial nerve, just posterior/posterior-medial to the knee. The ankle joint was secured at a 90° angle. The peak isometric torque was achieved by varying the current delivered to the nerve at a frequency of 100 Hz, 0.1-ms square wave pulse, 500 ms stimulation duration. Force frequency data was recorded by stimulating using frequencies ranging from 1 to 150 Hz with 30 s recovery between each measurement. For relative force measurements, force of the same animal was measured before and after treatment, and the percent change was calculated. Force measurement acquisition was blinded where researchers were unaware of the genotype or treatment conditions. Data were collected with the Aurora Scientific Dynamic Muscle Data Acquisition and Analysis Software.

### RNA-Seq

For RNA-seq, Integrin α7+CD34+Lin− MuSCs were isolated as described above. RNA was isolated using Qiagen RNAEasy Micro kit from 5,000–10,000 cells and cDNA generated and amplified using NuGEN Ovation RNA-Seq System v2 kit. Libraries were constructed from cDNA with the TruSEQ RNA Library Preparation Kit v2 (Illumina) and sequenced to 30–40×10^6^ × 75-bp reads per sample on a NextSeq 500 from the Stanford Functional Genomics Facility.

### RNA-Seq analysis

For the RNA-Seq analysis, bcbio-nextgen framework (https://bcbio-nextgen.readthedocs.io/) was used (version: 1.1.8-b): RNA sequences were aligned against the Mus musculus genome (mm10) using STAR^68^. RSEM^69^ or Salmon^70^ was used for calling transcripts and calculating transcripts per million (TPM) values as well as total counts. A counts matrix containing the number of counts for each gene and each sample was obtained. This matrix was analyzed by DESeq to calculate statistical analysis of significance^71^ of genes between samples. Pathway analysis of differentially expressed genes was analyzed using Ingenuity pathway analysis (QIAGEN IPA, https://digitalinsights.qiagen.com/IPA)^72^.

### Statistics

A minimum of three independent experiments (or animals) were used for all assays. Statistical analyses were performed using GraphPad Prism. We used a paired *t* test for experiments where control samples were from the same experiment in vitro. A non-parametric Mann–Whitney test was used to determine the significance difference between control vs experimental or untreated vs treated groups using α=0.05. ANOVA was performed for multiple comparisons with significance level determined using Fisher’s LSD test as indicated in the figure legends. Unless otherwise described, data are shown as the mean ± s.e.m.

### Reporting summary

Further information on research design is available in the Nature Research Reporting Summary linked to this article.

## Supplementary information

Supplementary Information

Reporting Summary

Description of Additional Supplementary Files

Supplementary Data 1

## Data Availability

All data are available in the main text or the supplementary materials. The RNAseq data generated in this study have been deposited in Gene Expression Omnibus (GEO) database under accession codes: GSE145297; GSE145312. Correspondence and requests for materials should be addressed to H.M.B. Source data are provided with this paper.

## Source data

Source data
