# Supplementary material for: Primary cilia on muscle stem cells are critical to maintain regenerative capacity and are lost during aging
Source: Nat Commun. 2022 Mar 17;13:1439. doi: 10.1038/s41467-022-29150-6 (PMC8931095; doi:10.1038/s41467-022-29150-6)
Supplement: Supplementary file 1 — Supplementary Information [file 41467_2022_29150_MOESM1_ESM.pdf]

Supplementary Materials for

Ciliation of muscle stem cells is critical to maintain regenerative capacity and is lost during aging

Adelaida R. Palla[1†], Keren I. Hilgendorf[2,3†], Ann V. Yang[1], Jaclyn P. Kerr[4], Aaron C. Hinken[4], Janos Demeter[2], Peggy Kraft[1], Nancie A. Mooney[2], Nora Yucel[1], David M. Burns[1], Yu Xin Wang[1], Peter K. Jackson[2, 5]* and Helen M. Blau[1]*

Correspondence to: hblau@stanford.edu, pjackson@stanford.edu

This PDF file includes:

Supplementary Figures 1-5

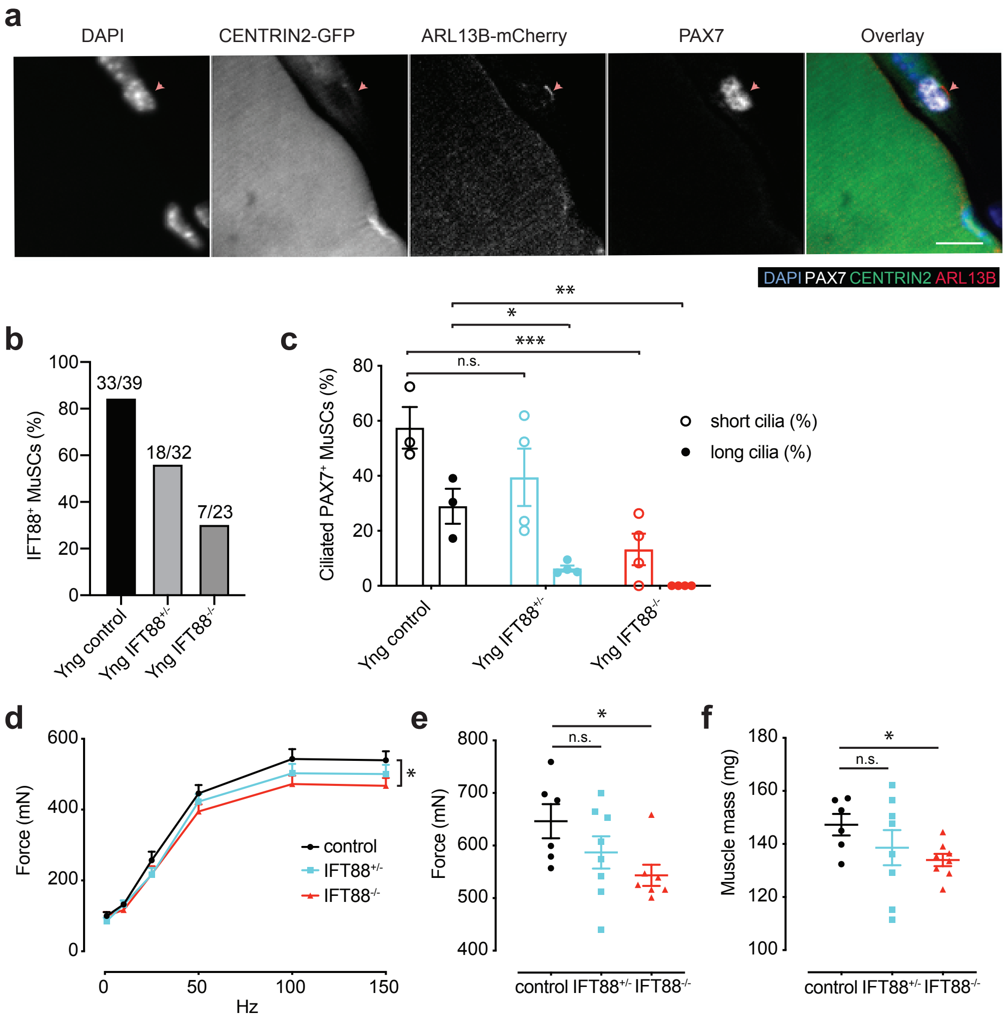

a
DAPI
CENTRIN2-GFP
ARL13B-mCherry
PAX7
Overlay
DAPI PAX7 CENTRIN2 ARL13B
b
IFT88+ MuSCs (%)
33/39
18/32
7/23
Yng control
Yng IFT88+/-
Yng IFT88-/-
c
Ciliated PAX7+ MuSCs (%)
n.s.
***
*
**
short cilia (%)
long cilia (%)
Yng control
Yng IFT88+/-
Yng IFT88-/-
d
Force (mN)
Hz
control
IFT88+/-
IFT88-/-
*
e
Force (mN)
n.s.
*
control IFT88+/- IFT88-/-
f
Muscle mass (mg)
n.s.
*
control IFT88+/- IFT88-/-

Supplementary Figure 1. Loss of cilia in MuSCs impairs muscle regeneration and strength recovery

(a) Myofibers were isolated from *Extensor digitorum longus* (EDL) muscles from transgenic CENTRIN2-GFP, ARL13B-mCherry mice. PAX7 staining was performed to identify MuSCs. All cells have a centrosome (CENTRIN2-GFP^{+}) and ciliated cells are also ARL13B-mCherry^{+}. Pink arrowhead indicates the primary cilium on the surface of MuSCs. Scale bars: 10 µM. **(b)** Pax7-specific Ift88 conditional knockout mice (*$Pax7^{CreERT2}$;$IFT88^{-/-}$*, IFT88$^{-/-}$*)*, heterozygous mice (*$Pax7^{CreERT2}$;$IFT88^{+/f}$*, IFT88$^{+/-}$*)* or control littermates (*$Pax7^{CreERT2}$;$IFT88^{+/+}$,* control*)* were treated with tamoxifen (TAM) at 8 weeks of age. Myofibers were isolated and stained for PAX7, FOP and IFT88. Percent of IFT88^{+} cilia on Pax7+ MuSCs was quantified (n=39 control, n=32 IFT88$^{+/-}$ and n=23 IFT88$^{-/-}$ myofibers were analyzed). **(c)** Percent of short (<1 µM) and long (>1 µM) cilia on Pax7+ MuSCs quantified from isolated myofibers of control, IFT88$^{+/-}$ and IFT88$^{-/-}$ mice (n=75 control, 79 IFT88$^{+/-}$, 81 IFT88$^{-/-}$ total myofibers were analyzed from 3 control and 4 IFT88$^{+/-}$ and IFT88$^{-/-}$ independent mice; average percent of ciliated MuSCs per mouse is shown). Individual replicates of Fig. 1c. **(d)** Force-frequency curves of control, IFT88$^{+/-}$ and IFT88$^{-/-}$ mice 2 weeks post-injury. **(e)** Plantar flexion tetanic isometric force of control, IFT88$^{+/-}$ and IFT88$^{-/-}$ mice on day 14 post-injury (absolute levels). **(f)** Gastrocnemius muscle mass 2 weeks post-injury. *P<0.05, **P<0.01, ***P<0.001. ANOVA test with Fisher's LSD for multiple comparisons (**c-f**). Source data are provided as a Source Data file. Means+s.e.m. n.s., non-significant.

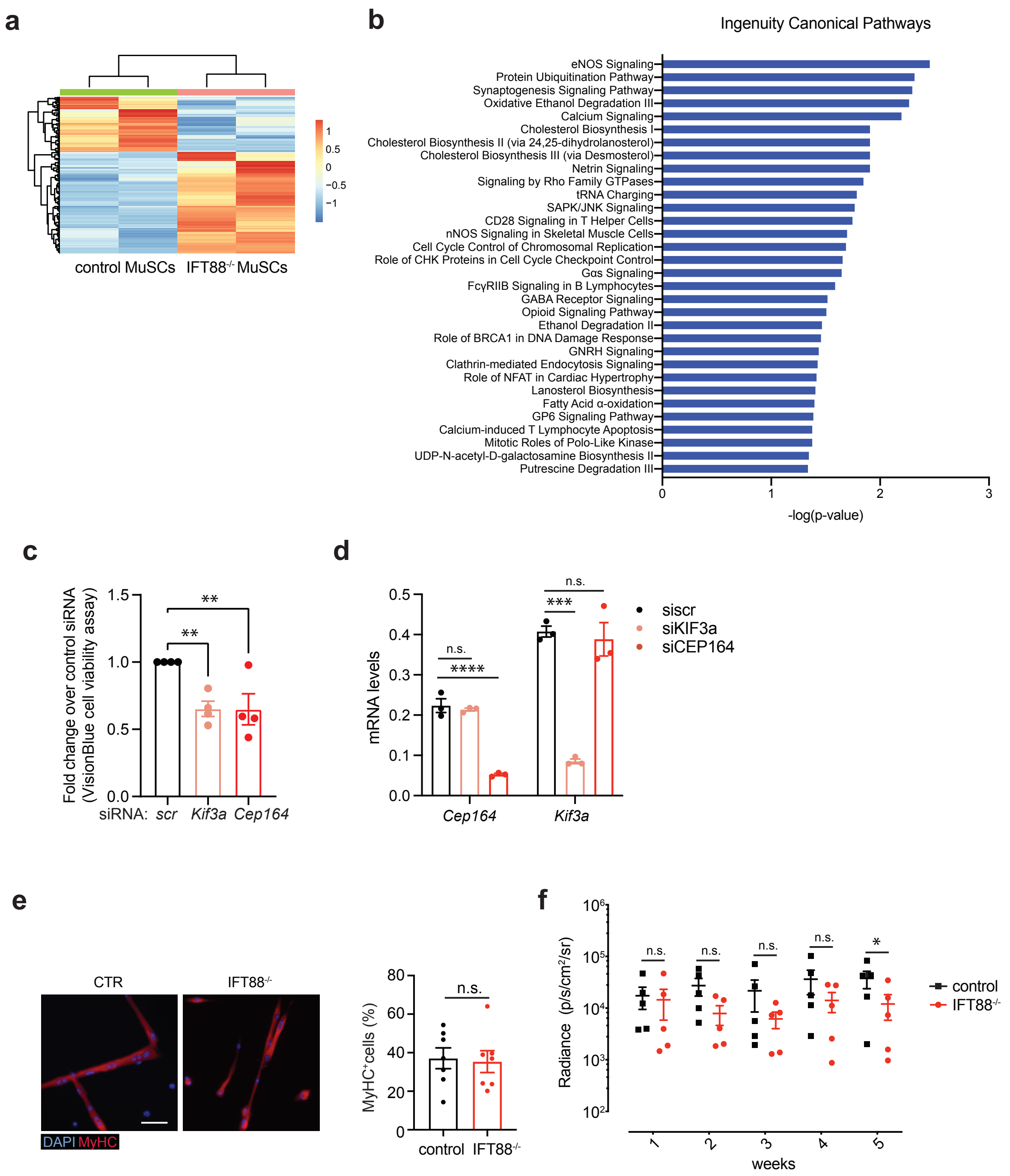
a
control MuSCs
IFT88-/- MuSCs
1
0.5
0
−0.5
−1
b
Ingenuity Canonical Pathways
eNOS Signaling
Protein Ubiquitination Pathway
Synaptogenesis Signaling Pathway
Oxidative Ethanol Degradation III
Calcium Signaling
Cholesterol Biosynthesis I
Cholesterol Biosynthesis II (via 24,25-dihydrolanosterol)
Cholesterol Biosynthesis III (via Desmosterol)
Netrin Signaling
Signaling by Rho Family GTPases
tRNA Charging
SAPK/JNK Signaling
CD28 Signaling in T Helper Cells
nNOS Signaling in Skeletal Muscle Cells
Cell Cycle Control of Chromosomal Replication
Role of CHK Proteins in Cell Cycle Checkpoint Control
Gαs Signaling
FcγRIIB Signaling in B Lymphocytes
GABA Receptor Signaling
Opioid Signaling Pathway
Ethanol Degradation II
Role of BRCA1 in DNA Damage Response
GNRH Signaling
Clathrin-mediated Endocytosis Signaling
Role of NFAT in Cardiac Hypertrophy
Lanosterol Biosynthesis
Fatty Acid α-oxidation
GP6 Signaling Pathway
Calcium-induced T Lymphocyte Apoptosis
Mitotic Roles of Polo-Like Kinase
UDP-N-acetyl-D-galactosamine Biosynthesis II
Putrescine Degradation III
0 1 2 3
-log(p-value)
c
Fold change over control siRNA (VisionBlue cell viability assay)
1.5 1.0 0.5 0.0
**
**
siRNA: scr Kif3a Cep164
d
mRNA levels
0.5 0.4 0.3 0.2 0.1 0.0
n.s.
****
n.s.
***
Cep164 Kif3a
siscr
siKIF3a
siCEP164
e
CTR
IFT88-/-
DAPI MyHC
MyHC+cells (%)
80 60 40 20 0
n.s.
control IFT88-/-
f
Radiance (p/s/cm²/sr)
10^6 10^5 10^4 10^3 10^2
n.s. n.s. n.s. n.s. *
1 2 3 4 5
weeks
control
IFT88-/-

Supplementary Figure 2. Transcriptome analysis of control and IFT88$^{-/-}$ MuSCs

(a) Heat map of differentially expressed genes (p<0.1) between control or IFT88$^{-/-}$ MuSCs freshly isolated from hindlimb muscles. **(b)** Enriched canonical pathways of the differentially expressed genes in IFT88$^{-/-}$ MuSCs indicated by Ingenuity Pathway Analysis (IPA). **(c)** Proliferation of MuSCs electroporated with siRNA targeting ciliary genes Kif3a, Cep164 or control (scrambled, scr), shown as fold change normalized to scr (n=4 independent mice averaged with 3 technical replicates each). **(d)** Gene expression levels of *Cep164* and *Kif3a* with siRNA targeting ciliary genes Kif3a, Cep164 or control (scrambled, scr) (n=3). **(e)** Left: Representative images of cultured control and IFT88$^{-/-}$ MuSCs after 7 days on collagen-coated plates showing MyHC immunostaining. Scale bars: 50 μM. Right: Quantification of MyHC positive control and IFT88$^{-/-}$ differentiated MuSCs (n=7 mice per genotype). **(f)** Individual replicates of Fig. 2a. Engraftment of GFP/luc-labeled control and IFT88$^{-/-}$ MuSCs. Bioluminescence imaging (BLI) signal post-transplant expressed as average radiance (p s^{-1} cm^{-2} sr^{-1}) (n=5 replicates per condition). ANOVA test with Fisher's LSD for multiple comparisons **(c,d,f)**; Mann Whitney test **(e)**. *P<0.05. Source data are provided as a Source Data file. Means+s.e.m. n.s., non-significant.

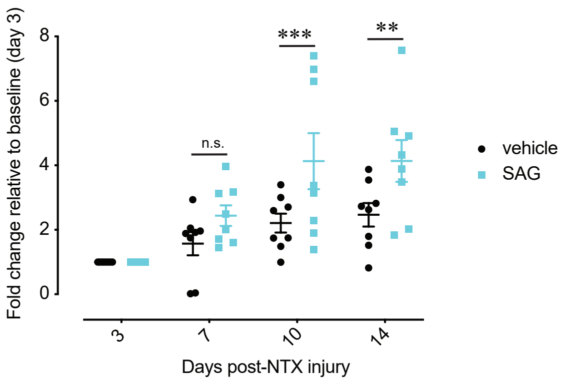

Supplementary Figure 3. SMO agonist treatment promotes MuSC expansion

Individual replicates of Fig. 3f. Expansion of endogenous MuSCs in Pax7^{CreERT2};Rosa26-LSL-Luc mice treated with tamoxifen (TAM) to label resting MuSCs and assayed by bioluminescence imaging (BLI) post-notexin injury. BLI signal was normalized to day 3 post-injury prior to vehicle or SAG1.3 injection (n = 8 mice per condition). **P<0.01, ***P<0.001. ANOVA test with Fisher's LSD for multiple comparisons. Source data are provided as a Source Data file. Means+s.e.m. n.s., non-significant.

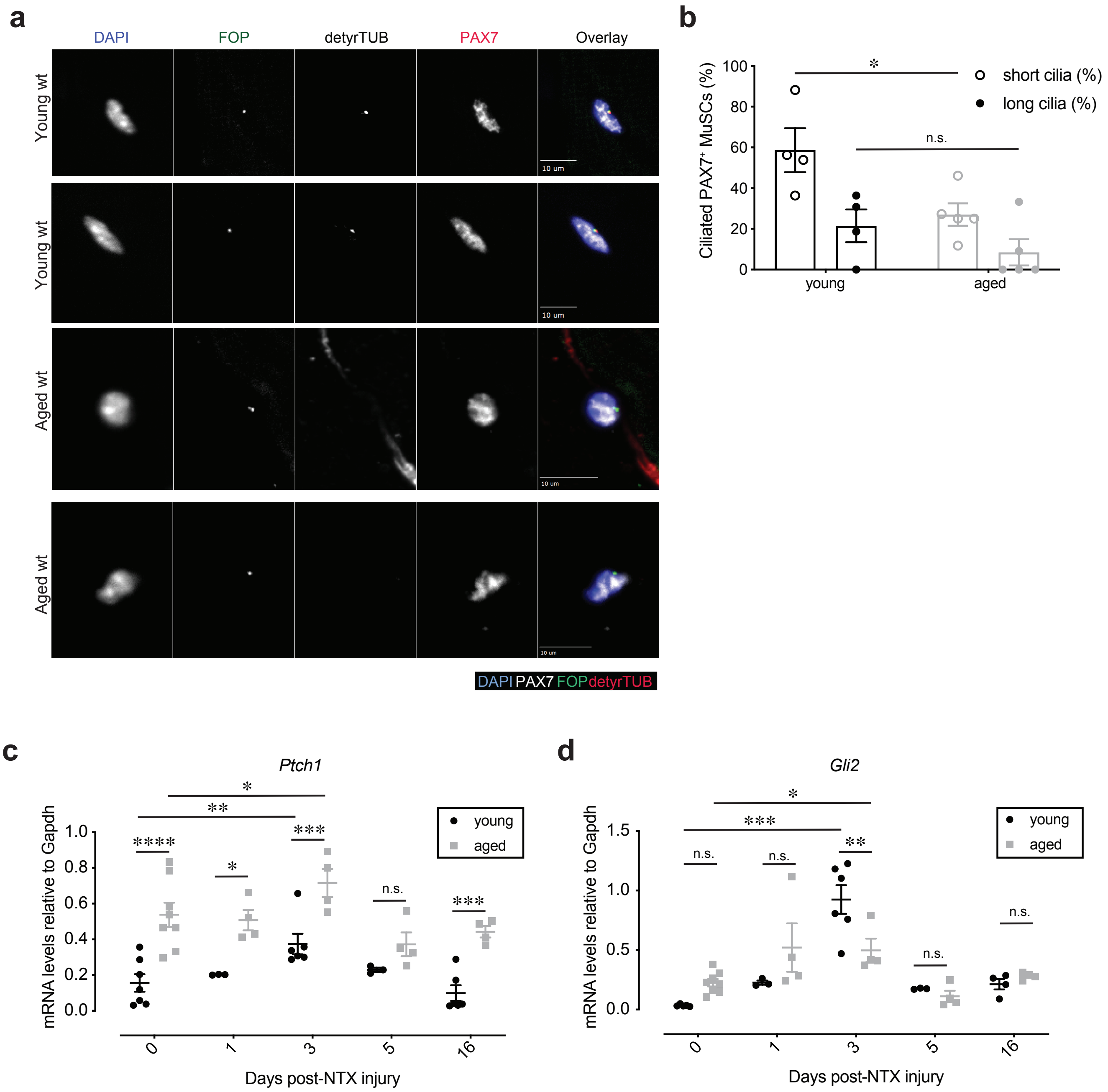

a
DAPI
FOP
detyrTUB
PAX7
Overlay
Young wt
Young wt
Aged wt
Aged wt
10 um
DAPI PAX7 FOP detyrTUB
b
Ciliated PAX7+ MuSCs (%)
100
80
60
40
20
0
*
n.s.
short cilia (%)
long cilia (%)
young
aged
c
Ptch1
mRNA levels relative to Gapdh
1.0
0.8
0.6
0.4
0.2
0.0
****
*
***
n.s.
***
**
*
young
aged
0
1
3
5
16
Days post-NTX injury
d
Gli2
mRNA levels relative to Gapdh
1.5
1.0
0.5
0.0
n.s.
n.s.
**
n.s.
n.s.
***
*
young
aged
0
1
3
5
16
Days post-NTX injury

Supplementary Figure 4. Aged MuSCs present decreased ciliation and Hedgehog signaling

(a) Representative confocal images of uninjured/resting *Extensor digitorum longus* (EDL) myofibers of young (2 months) and aged (>24 months) C57BL/6 wild-type (wt) mice showing cilia immunostaining in $Pax7^+$ MuSCs. Scale bars: 10 µm. DAPI, blue; PAX7, white; FOP, green; detyrosinated tubulin, red. **(b)** Percent of short (<1 µM) and long (>1 µM) cilia on Pax7+ MuSCs quantified isolated myofibers from young and aged myofibers (n=58 myofibers total per age group isolated from n=4 young and n=5 aged mice, average percent of ciliated MuSCs per mouse is shown). **(c)** Individual replicates for Fig. 4e. Expression of *Ptch1* after *Tibialis anterior* muscle injury. n=3-7 mice per timepoint. **(d)** Individual replicates for Fig. 4f. Expression of *Gli2* after *Tibialis anterior* muscle injury. n=3-7 mice per timepoint. ANOVA test with Fisher's LSD for multiple comparisons **(b-d).** *P<0.05, **P<0.01, ***P<0.001 ****P<0.0001. Source data are provided as a Source Data file. Mean + SEM. n.s., non-significant.

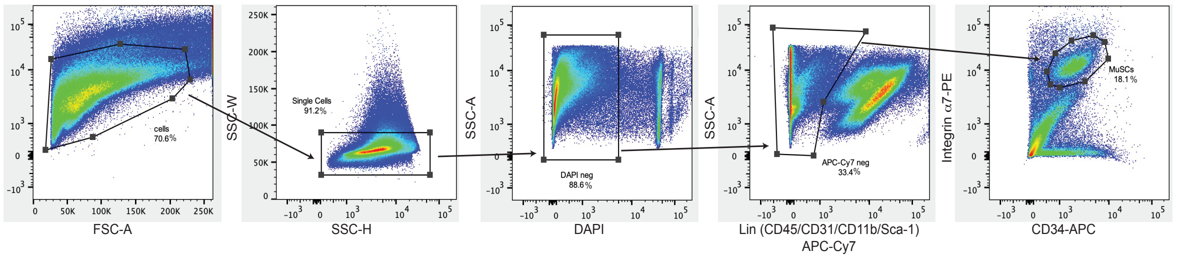

Supplementary Figure 5. Muscle Stem Cell Sorting Strategy

Compensation was performed due to overlap between APC and APC-Cy7. SSC and FSC were gated for small cells. Cells were gated for DAPI negative, Lin-APC-Cy7 negative (CD31, SCA1, CD45, CD11B), a7-PE positive, and CD34-APC positive.
