# Supplementary material for: Primary cilia on muscle stem cells are critical to maintain regenerative capacity and are lost during aging
Source: Nat Commun. 2022 Mar 17;13:1439. doi: 10.1038/s41467-022-29150-6 (PMC8931095; doi:10.1038/s41467-022-29150-6)
Supplement: Supplementary file 2 — Reporting Summary [file 41467_2022_29150_MOESM2_ESM.pdf]

nature research

Corresponding author(s): Helen M. Blau; Peter K. Jackson

Last updated by author(s): Jan 18, 2022

Reporting Summary

Nature Research wishes to improve the reproducibility of the work that we publish. This form provides structure for consistency and transparency in reporting. For further information on Nature Research policies, see our Editorial Policies and the Editorial Policy Checklist.

Statistics

For all statistical analyses, confirm that the following items are present in the figure legend, table legend, main text, or Methods section.

n/a	Confirmed	
☐	☒	The exact sample size (n) for each experimental group/condition, given as a discrete number and unit of measurement
☐	☒	A statement on whether measurements were taken from distinct samples or whether the same sample was measured repeatedly
☐	☒	The statistical test(s) used AND whether they are one- or two-sided<br>*Only common tests should be described solely by name; describe more complex techniques in the Methods section.*
☒	☐	A description of all covariates tested
☐	☒	A description of any assumptions or corrections, such as tests of normality and adjustment for multiple comparisons
☐	☒	A full description of the statistical parameters including central tendency (e.g. means) or other basic estimates (e.g. regression coefficient) AND variation (e.g. standard deviation) or associated estimates of uncertainty (e.g. confidence intervals)
☐	☒	For null hypothesis testing, the test statistic (e.g. F, t, r) with confidence intervals, effect sizes, degrees of freedom and P value noted<br>*Give P values as exact values whenever suitable.*
☒	☐	For Bayesian analysis, information on the choice of priors and Markov chain Monte Carlo settings
☒	☐	For hierarchical and complex designs, identification of the appropriate level for tests and full reporting of outcomes
☒	☐	Estimates of effect sizes (e.g. Cohen's d, Pearson's r), indicating how they were calculated

Our web collection on statistics for biologists contains articles on many of the points above.

Software and code

Policy information about availability of computer code

Data collection	Muscle force measurements were acquired using the DMC software (Aurora Scientific). Confocal images of myofibers were acquired using the Image Acquisition Software SlideBook 6.0 (3i). VisionBlue was acquired with the fluorescence plate reader (Infinite M1000 PRO, Tecan) using the Magellan software (Tecan). Bioluminescent images from mice were acquired using the Living Image software (Perkin Elmer). RNAseq was performed using the NextSeq 550 (Illumina) and Ilumina Casava1.7 software was used for basecalling. Muscle transverse sections images were acquired on a Keyence BZ-X700 all-in-one fluorescence microscope (Keyence) with the Keyence Image Acquisition Software BX-H3XD (Keyence). FACS data collected with the FACSDiva software (BD Biosciences). qPCR data was acquired using the Applied Biosystems 7900HT Real-Time PCR System Software (Thermo Fisher Scientific).
Data analysis	Bioluminescent images were analyzed using the Living Image software (Perkin Elmer). Muscle force measurements were analyzed using the DMA software (Aurora Scientific). Muscle transverse section images were analyzed using the BZ-H3AE Analysis Software (Keyence) to segment the fibers in the image to analyze the area of each fiber. RNA-Seq analysis used the following softwares: bcbio-nextgen framework (https://bcbio-nextgen.readthedocs.io/) (version: 1.1.8-b) was used, RNA sequences were aligned against the Mus musculus genome (mm10) using STAR. RSEM or Salmon was used for calling transcripts and calculating transcripts per million (TPM) values as well as total counts. DESeq was used to calculate statistical analysis of significance of genes between samples. Quantification and length of the primary cilium of immunofluorescent images were analyzed using ImageJ. FACS data were analyzed with FlowJo software (FlowJo v10.0, BD). Statistical analyses were performed using GraphPad Prism.

For manuscripts utilizing custom algorithms or software that are central to the research but not yet described in published literature, software must be made available to editors and reviewers. We strongly encourage code deposition in a community repository (e.g. GitHub). See the Nature Research guidelines for submitting code & software for further information.

Data

Policy information about availability of data

All manuscripts must include a data availability statement. This statement should provide the following information, where applicable:

- Accession codes, unique identifiers, or web links for publicly available datasets
- A list of figures that have associated raw data
- A description of any restrictions on data availability

The RNA-seq data used in this study are available in the Gene Expression Omnibus database under the accession codes GSE145297 [https://www.ncbi.nlm.nih.gov/geo/query/acc.cgi?acc=GSE145297]; GSE145312 [https://www.ncbi.nlm.nih.gov/geo/query/acc.cgi?acc=GSE145312]. Source data are provided with this paper.

Field-specific reporting

Please select the one below that is the best fit for your research. If you are not sure, read the appropriate sections before making your selection.

[x] Life sciences [ ] Behavioural & social sciences [ ] Ecological, evolutionary & environmental sciences

For a reference copy of the document with all sections, see nature.com/documents/nr-reporting-summary-flat.pdf

Life sciences study design

All studies must disclose on these points even when the disclosure is negative.

Sample size	No statistical methods were used to determine the sample size. The sample size was chosen based on historical data or similar published studies. Please see: Sacco et al., 2008, Cosgrove et al., 2014 and Ho et al., 2017.
Data exclusions	Mice that developed dermatitis throughout the treatment period or with visible tumors observed at the endpoint necropsy were excluded from the study.
Replication	A minimum of three independent experiments (or animals) were used for all assays. The exact number of replicates in groups or independent experiments is mentioned in the figure legends or methods sections.
Randomization	When mice were allocated to their experimental groups comparing their genotype or age group, there was no randomization. Animals of the same genotype or age group were randomly allocated to either treated or non-treated groups.
Blinding	For imaging acquisition, data analyses were blinded. The researchers performing the imaging acquisition and scoring were unaware of conditions given to sample groups analyzed. The genotype or treatment was decoded after acquisition and analysis.<br><br>For in vivo force measurements and bioluminescence imaging acquisition and analysis, investigators were blinded to the genotype and/or treatment conditions.<br><br>For cell culture and sorting studies (proliferation assays, qPCR, sequencing and flow cytometry) were not blinded but carried out using standard procedures that should not cause biased results.

Reporting for specific materials, systems and methods

We require information from authors about some types of materials, experimental systems and methods used in many studies. Here, indicate whether each material, system or method listed is relevant to your study. If you are not sure if a list item applies to your research, read the appropriate section before selecting a response.

Materials & experimental systems

n/a	Involved in the study	
[ ]	[x]	Antibodies
[x]	[ ]	Eukaryotic cell lines
[x]	[ ]	Palaeontology and archaeology
[ ]	[x]	Animals and other organisms
[x]	[ ]	Human research participants
[x]	[ ]	Clinical data
[x]	[ ]	Dual use research of concern

Methods

n/a	Involved in the study	
[x]	[ ]	ChIP-seq
[ ]	[x]	Flow cytometry
[x]	[ ]	MRI-based neuroimaging

Antibodies

Antibodies used	Biotin anti-CD45: BD Biosciences, clone 30F11, catalog # 553078<br>Biotin anti-CD11b: BD Biosciences, clone M1/70, catalog # 553309<br>Biotin anti-CD31: eBioscience, clone 390, catalog # 13-0311-82<br>Biotin anti-Sca1 (Ly-6A/E): BD Biosciences clone E13-161.7, catalog # 553334<br>anti-Mouse CD34 eFluor 660: eBioscience, clone RAM34, catalog # 50-0341-82<br>anti-integrin alpha 7 antibody-PE conjugate: Ablab, clone R2F2, catalog # 10ST215<br>anti-detyrosinated tubulin abcam, catalog # ab48389<br>anti-IFT88: ProteinTech, catalog # 13967-1-ap<br>anti-PAX7: Santa Cruz Biotechnology, catalog # sc-81648<br>anti-FOP: Abnova, clone 2B1, catalog #H00011116-M01<br>anti-MyHC: Thermo Fisher Scientific, clone MF20, catalog # 14-6503-82<br>anti-LAMININ: Millipore, clone A5, catalog # 05-206
Validation	Commercially available and well-established antibodies were used. All antibodies have been tested for reactivity against the appropriate species on the specification sheets on the providers' websites or in published articles. The muscle stem cell antibody sorting panel (anti-CD45: BD Biosciences catalog # 553078; anti-CD11b: BD Biosciences catalog # 553309; anti-Sca1 (Ly-6A/E): BD Biosciences catalog # 553334; anti-CD31: eBioscience catalog # 13-0311-82; anti-Mouse CD34 eFluor 660: eBioscience catalog # 50-0341-82; anti-alpha 7 integrin antibody-PE conjugate: Ablab, catalog # 10ST215) has been validated previously (Sacco et al., 2008; Cosgrove et al., 2014; Ho et al., 2017). Anti-detyrosinated tubulin (abcam, catalog # ab48389) recognizes a 10-residue synthetic peptide of the C terminal domain of human alpha Tubulin. It has been validated previously to detect the primary cilium in cells as shown in the manufacturer's website. Anti-PAX7 (Santa Cruz Biotechnology, catalog # sc-81648) detects Pax-7 of mouse, rat, human and avian origin by WB, IP and IF and has been validated for detection of muscle stem cells as shown in the manufacturer's website. anti-FOP (Abnova, catalog #H00011116-M01) detects FGFR1OP in mouse by IF reported in scientific literature (PMID: 28625565) and validated by the manufacturer. Anti-MyHC MF20 (Thermo Fisher Scientific, catalog # 14-6503-82) recognizes the heavy chain of myosin II, specifically the light meromyosin portion, in cardiac and skeletal muscle of vertebrates. The MF20 antibody has been shown to react to myosin from a variety of mammalian, avian and amphibian species, including rat, mouse, human, chicken, zebrafish, and dog; and has been reported for use in western blotting, and immunocytochemistry. anti-LAMININ: Millipore, clone A5, catalog # 05-206 specifically detects B2 chain laminin; does not cross-react with other basement membrane components or fibronectin. It recognizes human, mouse and monkey and reported for use in immunocytochemistry.

Animals and other organisms

Policy information about studies involving animals; ARRIVE guidelines recommended for reporting animal research

Laboratory animals	C57Bl/6J wt mice: Male young (2-4 months) and male aged (>24 months)<br>NOD-SCID mice: 2-4 months, male and female<br>Pax7CreERT2;IFT88flox/flox: young (2-4 months, male); aged (12 months, male)<br>Pax7CreERT2;Rosa26-LSL-Luc: 2-4 months, male and female<br>Centrin2-eGFP; Arl13b-mCherry: 2-4 months, male
Wild animals	No wild animals were used in this study.
Field-collected samples	No field collected samples were used in this study
Ethics oversight	All experiments and protocols were performed in compliance with the institutional guidelines of Stanford University and Administrative Panel on Laboratory Animal Care (APLAC). The laboratory animal care program at Stanford University is accredited by the Association for the Assessment and Accreditation of Laboratory Animal Care (AAALAC International). All studies were conducted in accordance with the GSK Policy on the Care, Welfare and Treatment of Laboratory Animals and were reviewed the Institutional Animal Care and Use Committee either at GSK or by the ethical review process at the institution where the work was performed.

Note that full information on the approval of the study protocol must also be provided in the manuscript.

Flow Cytometry

Plots

Confirm that:

- [x] The axis labels state the marker and fluorochrome used (e.g. CD4-FITC).
- [x] The axis scales are clearly visible. Include numbers along axes only for bottom left plot of group (a 'group' is an analysis of identical markers).
- [x] All plots are contour plots with outliers or pseudocolor plots.
- [x] A numerical value for number of cells or percentage (with statistics) is provided.

Methodology

Sample preparation	Hindlimb muscles from mice were minced and digested using a collagenase type 2 (Worthington Biochemical, cat # LS004177) and dispase II (Thermo Fisher Scientific, cat # 17105041) solution by the gentleMACs Octo Dissociator (Miltenyi) at 37ºC. Homogenized muscles were passed through 40 uM nylon strainers to obtain a single cell suspension. Subsequently, single cells were depleted for hematopoietic lineage expressing and non-muscle cells (CD45-/CD11b-//Sca1-/CD31-) (BD Biosciences catalog # 553078, 553309, 553334 and eBioscience 13-0311-82 respectively) using a magnetic bead column (Miltenyi). The remaining Lin- cell mixture was then subjected to FACS analysis to sort for MuSCs co-expressing CD34 (Anti-Mouse CD34 eFluor 660, eBioscience, cat# 50-0341-82) and integrin alpha7 (Anti-integrin a7 antibody-PE conjugate, Ablabs) markers.
Instrument	FACS LSR II (BD Biosciences)
Software	FACSDIVA Software (BD Biosciences)
Cell population abundance	Cells were not post-sorted after initial purity sorting.
Gating strategy	Muscle stem cell sort from muscle tissues:<br>FSC and SSC were used to identify muscle stem cells and exclude doublets or debris. After gating for live cells (DAPI-), lineage negative cells were excluded (Cd11b-, Cd45-, Cd31-, Sca1-). Then, muscle stem cells were identified as the double positive integrin alpha7+ and Cd34+ in the Lin- population.

☒ Tick this box to confirm that a figure exemplifying the gating strategy is provided in the Supplementary Information.
