# Supplementary material for: Primary cilia on muscle stem cells are critical to maintain regenerative capacity and are lost during aging
Source: Nat Commun. 2022 Mar 17;13:1439. doi: 10.1038/s41467-022-29150-6 (PMC8931095; doi:10.1038/s41467-022-29150-6)
Supplement: Supplementary file 3 — Description of Additional Supplementary Files [file 41467_2022_29150_MOESM3_ESM.pdf]

Title: Supplementary Data 1:
Description: RNAseq analysis of control and IFT88$^{-/-}$ muscle stem cells. Differentially expressed genes between control and IFT88$^{-/-}$ MuSCs were analyzed through the use of Ingenuity pathway analysis (QIAGEN IPA, https://digitalinsights.qiagen.com/IPA).
